# Prediction of the Maturity of Greenhouse Grapes Based on Imaging Technology

**DOI:** 10.34133/2022/9753427

**Published:** 2022-03-30

**Authors:** Xinguang Wei, Linlin Wu, Dong Ge, Mingze Yao, Yikui Bai

**Affiliations:** ^1^College of Water Conservancy, Shenyang Agricultural University, Shenyang 110866, China; ^2^Institute of Soil and Water Conservation, Northwest A&F University, 712100, Yangling, Shaanxi Province, China

## Abstract

To predict grape maturity in solar greenhouses, a plant phenotype-monitoring platform (Phenofix, France) was used to obtain RGB images of grapes from expansion to maturity. Horizontal and longitudinal diameters, compactness, soluble solid content (SSC), titratable acid content, and the SSC/acid of grapes were measured and evaluated. The color values (*R*, *G*, *B*, *H*, *S*, and *I*) of the grape skin were determined and subjected to a back-propagation neural network algorithm (BPNN) to predict grape maturity. The results showed that the physical and chemical properties (PCP) of the three varieties of grapes changed significantly during the berry expansion stage and the color-changing maturity stage. According to the normalized rate of change of the PCP indicators, the ripening process of the three varieties of grapes could be divided into two stages: an immature stage (maturity coefficient Mc < 0.7) and a mature stage (after which color changes occurred) (0.7 ≤ Mc < 1). When predicting grape maturity based on the *R*, *G*, *B*, *H*, *I*, and *S* color values, the *R*, *G*, and *I* as well as *G*, *H*, and *I* performed well for Drunk Incense, Muscat Hamburg, and Xiang Yue grape maturity prediction. The GPI ranked in the top three (up to 0.87) when the above indicators were used in combination with BPNN to predict the grape Mc by single-factor and combined-factor analysis. The results showed that the prediction accuracy (RG and HI) of the two-factor combination was better for Drunk Incense, Muscat Hamburg, and Xiang Yue grapes (with recognition accuracies of 79.3%, 78.2%, and 79.4%, respectively), and all of the predictive values were higher than those of the single-factor predictions. Using a confusion matrix to compare the accuracy of the Mc's predictive ability under the two-factor combination method, the prediction accuracies were in the following order: Xiang Yue (88%) > Muscat Hamburg (81.3%) > Drunk Incense (76%). The results of this study provide an effective way to predict the ripeness of grapes in the greenhouse.

## 1. Introduction

In the process of crop growth, it is very important for crop managers to obtain information on fruit maturity time and yield [1]. Traditional crop characteristic determination and yield measurements are carried out by destructive sampling in the field, which is time-consuming, laborious, and prone to large amounts of human error [2]. Therefore, it is necessary to explore a fast, accurate, and nondestructive method of maturity prediction [3]. In recent years, with the development of artificial intelligence, the development of greenhouse intelligence has increased, and an increasing number of greenhouses include dynamic monitoring that allows the determination of main environmental factors [4], growth factors [5], and phenotypic parameters [6] throughout the whole life cycle of greenhouse crops and the accurate determination of greenhouse crop biomass, quality, and other information; this in turn enables the determination of the growth dynamics of greenhouse crops, on which basis accurate regulatory and growth process controls can be implemented. Therefore, it is of great significance to improve the management of greenhouse crops and the quality and efficiency of the greenhouse production industry [7].

In recent years, with the support of imaging technology and machine vision technology, crop maturity prediction has gradually become a popular research topic [8–10]. Previous studies have used image technology to predict crop maturity, mainly via spectral information prediction [11], electronic noses, and electronic tongues [12] combined with partial least squares regression analysis and via color eigenvalues combined with back-propagation neural networks [13]. The BP neural network is considered a highly accurate model when the internal mechanism and relation are uncertain [14]. Among these methods, back-propagation neural networks (BPNNs) are multilayer neural networks that can be trained according to the error back-propagation, and these networks are the most widely used neural networks [15]. The use of BPNNs can improve the accuracy of prediction by considering the interaction between input and output, which makes them more effective evaluation models than traditional discriminant analysis and multivariate logistic regression area [16]. The majority of previous related studies used BPNNs for functional approximation, regression analysis, numerical prediction, classification, and data processing [17–19].

The computer vision technology was used to calculate color values of tomato fruits. To distinguish the ripeness of tomatoes, the correct recognition rate reached 90.8% [20]. Xiong et al. [21] studied the visual quality of immature, mature, and rotten litchi fruits after maturity. The color range of the Cr component in the YCbCr color space was used to determine the ripeness of the fruit, and the recognition accuracy reached 91%. The color rate of navel oranges was studied and established an artificial neural network model by taking the mean value and mean square error of the *H*, *S*, *R*, *G*, and *B* color components as color parameters [22]. The grading consistency rates of this model and the artificial standard were 90% and 92%, respectively. Image technology and machine vision technology can be used to predict crop maturity [23, 24]. Previous methods such as the use of color eigenvalues combined with artificial neural networks and partial least squares regression models have mainly been used. Researchers have mainly focused on field crop species, greenhouse fruits and vegetables, and other crop species. However, research on greenhouse fruit is less prevalent; research on greenhouse grape maturity prediction is especially relatively scarce.

There have been few systematic studies on grape maturity, especially on the relationship between grape skin color and grape ripeness in different ripening periods under the condition of solar greenhouse; on the other hand, the color eigenvalue parameters and their combination are different from the prediction methods in the previous research; there is no uniform and referable method to predict the maturity of grape. Therefore, this study was aimed at monitoring the growth of grapes during the growth period via a plant phenotype-monitoring platform; based on the back-propagation neural network, the first three factors of grape skin color characteristic value (*R*, *G*, *B*, *H*, *I*, and *S*) are combined with each other to predict the grape maturity in greenhouse, in order to provide reference for biomass monitoring, fruit picking, and greenhouse management in solar greenhouse.

## 2. Materials and Method

### 2.1. Study Site

This study area was a research greenhouse of Shenyang Agricultural University (41°49′N, 123°34′E, altitude 43 m above sea level), Liaoning, China. The greenhouse is a Liaoning Solar Greenhouse [25]. The study area has a temperate continental monsoon climate, with an annual sunshine duration of 2530 h and an annual average temperature of 8°C. The greenhouse faces south, with a length of 60 m, a width of 8 m, and a ridge height of 4 m. The greenhouse is covered with PO film to prevent rain and to provide thermal insulation. The tested soil was a clay loam, with a bulk soil compactness of 1.44 g/cm^3^ from a depth of 0 to 60 cm, a field water holding capacity of 22.3% (mass moisture content), and a wilting point of 9.0% (mass moisture content). During the study period, the greenhouse was not affected by severe climate disasters or pests.

### 2.2. Image Acquisition and Sample Collection

The grape varieties tested were Drunk Incense, Muscat Hamburg, and Xiang Yue. Both Drunk Incense and Xiang Yue were planted in March 2015, with a plant spacing of 0.5 m and a row spacing of 2.5 m, and Muscat Hamburg was planted in March 2016. Other greenhouse management practices were performed in accordance with the actual production practices of local greenhouse-produced grapes. In this study, the key growth periods, such as the swelling period and the color maturity period, of various grape varieties were selected for measurement. Ten clusters of grapes with uniform growth were selected for each variety, and a 20 cm × 40 cm whiteboard was used as a reference object on which the grapes were placed such that the same plane was used as the background of the grapes when the images were taken. The plant phenotype-monitoring platform (Phenofix, France) was parallel to the image in the shooting frame, and the crop name and shooting area number are inputted into the system software before shooting. Starting at 4 weeks after flowering and continuing to harvest, sampling was conducted twice a week. After the samples were collected, their physical properties were measured, and their quality properties were evaluated after the image analysis process.

### 2.3. Determination of Physical and Quality Characteristics of Grapes

The physical traits of the grapes were dynamically monitored beginning four weeks after flowering. The vertical and horizontal diameters of the grape berries were measured with a Vernier caliper, and the measurement frequency was every 7-10 days. ImageJ software was used to calculate the fruit boundary area (i.e., the longitudinal diameter×the horizontal diameter) and the actual fruit area to calculate the fruit compactness, that is, the fruit boundary area/the actual fruit area. After the physical indicators of the sample were measured, the chemical indicators of the samples were determined. The digital Abbe refractometer method [26] was used to determine the soluble solid content, and the 0.1 mol·L^−1^ NaOH titration method [27] was used to determine the titratable acid content.

### 2.4. Image Segmentation and Obtaining the Grape Skin Color Values

**Figure 1:**
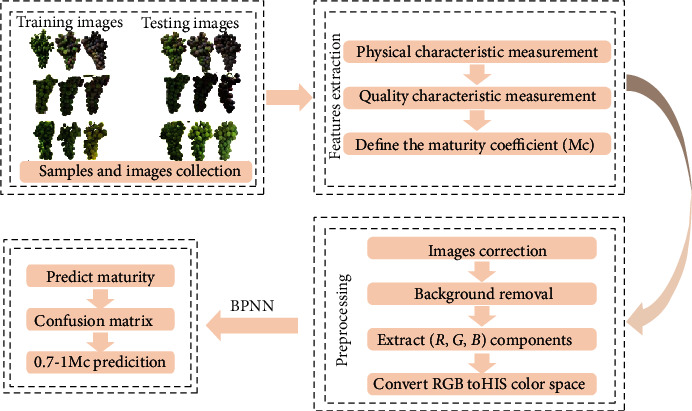
Flow chart of grape skin color feature value combined with back-propagation neural network (BPNN) for predicting grape maturity.

Initially, geometric correction of projection transformation was used to make geometric correction on the image. And then, the RGB images were segmented using an image processing method developed in MATLAB software (Version R2018a, MathWorks, USA). The images were processed to identify the region of interest (ROI) and remove background and objects that are not part of the grape. Segmentation was performed using Otsu method [28]. The flow of image preprocessing was shown in Figure 1.

Images of grape were acquired in the RGB color space. The RGB parameters extracted from the images were red color space (red), green color space (green), and blue color space (blue). RGB values are key indicators for determining the brightness of primary colors. The darker a color is, the lower the brightness, and the smaller the value. HIS represents the vividness of the color. Compared with the RGB color model, this method is more in line with the understanding of the human visual system. It is composed of hue (*H*), intensity, (*I*), and saturation (*S*) values. HIS values can be derived from the RGB color space, and the conversion formulas are as follows (where “min” represents the minimum *R*, *G*, and *B* value) [29, 30]:

(1) $$\begin{array}{c} I=\frac{R+G+B}{3}, \end{array}$$

(2) $$\begin{array}{c} S=1-\frac{3\min\left(R,G,B\right)}{R+G+B}, \end{array}$$

(3) $$\begin{array}{c} H=60*\frac{G-B}{\left(\max-\min\right)\text{ if }\left(R==\max\right)}, \end{array}$$

(4) $$\begin{array}{c} H=60*\frac{B-R}{\left(\max-\min\right)+120\text{ if }\left(G==\max\right)}, \end{array}$$

(5) $$\begin{array}{c} H=60*\frac{R-G}{\left(\max-\min\right)+240\text{ if }\left(B==\max\right)}. \end{array}$$

### 2.5. Prediction of Grape Maturity Level

The study used a back-propagation neural network (BPNN) to predict the maturity level of grape samples. The maturity coefficient (Mc) of the detection value was defined as 0.1-1 Mc, and samples for which the Mc ranged from 0.1-1 were selected for image analysis to determine the color of the grape skin. A feature value was used to construct a sample set of the neural network training set (70 samples of different varieties of grapes were collected), and the goal of the neural network training set was set to 0.1-1.40. Other grape samples at three different maturity levels were selected to obtain the values of the skin color to establish the verification set of the neural network. The number of neurons in the hidden layer was determined by using MATLAB 2017a software and on the basis of the accuracy of the test results. The recognition function of the neural network was defined as a logarithmic function [31]:

(6) $$\begin{array}{c} f\left(x\right)=\frac{1}{1+\exp\left(-x\right)}. \end{array}$$

### 2.6. Model Accuracy and Performance Analysis

The coefficient of determination (*R*^2^), root mean square error (RMSE), consistency index (*d*) (the value ranged from 0~1; a larger value indicates a higher degree of consistency between the measured value and the predicted value), average absolute error (MAE), and the overall evaluation index (GPI) were used to evaluate the estimation effect of the grape maturity prediction model. The model accuracy and performance were evaluated under the following criteria [32]:

(7) $$\begin{array}{c} R^{2}=\frac{\sum_{i=1}^{n}{\left(X_{i}-\overset{¯}{X}\right)}^{2}{\left(Y_{i}-\overset{¯}{Y}\right)}^{2}}{\sum_{i=1}^{n}{\left(X_{i}-\overset{¯}{X}\right)}^{2}\sum_{i=1}^{n}{\left(Y_{i}-\overset{¯}{Y}\right)}^{2}},\\ RMSE=\sqrt{\frac{\sum_{i=1}^{n}{\left(Y_{i}-X_{i}\right)}^{2}}{n-1},}\\ d=1-\frac{\sum_{i=1}^{n}{\left(X_{i}-Y_{i}\right)}^{2}}{\sum_{i=0}^{n}{\left(\left|Y_{i}-\overset{¯}{Y}\right|+\left|X_{i}-\overset{¯}{X}\right|\right)}^{2}},\\ \text{MAE}=\frac{\sum_{i=1}^{n}\left|Xi-Yi\right|}{n},\\ \text{GPI}=\overset{4}{\underset{j=1}{\sum }}\left[\alpha \left(\overset{¯}{O_{j}}-O_{j}\right)\right], \end{array}$$

where *X*_*i*_ and $\;\overset{¯}{X}$ are the actual measured value and the mean of the measured value, respectively; *Y*_*i*_ and *Y* are the estimated value and the mean of the estimated value, respectively; *n* is the sample number of the estimated model; *O*_*j*_ is the normalized value of the above mentioned four evaluation indicators (*R*^2^, RMSE, *d*, and MAE) [25]; and $\overset{¯}{O_{j}}$ is the normalized median of the index *j* of the 6 models. When *j* is the MAE and RMSE, *α* = 1. When *j* is the *R*^2^ and *d*, *α* = −1. The higher the GPI is, the better the overall prediction effect. The prediction effect was presented in the form of ranking. The higher the ranking is, the better the prediction effect.

## 3. Results

### 3.1. Physical and Chemical Properties of Grapes of Different Varieties

**Figure 2:**
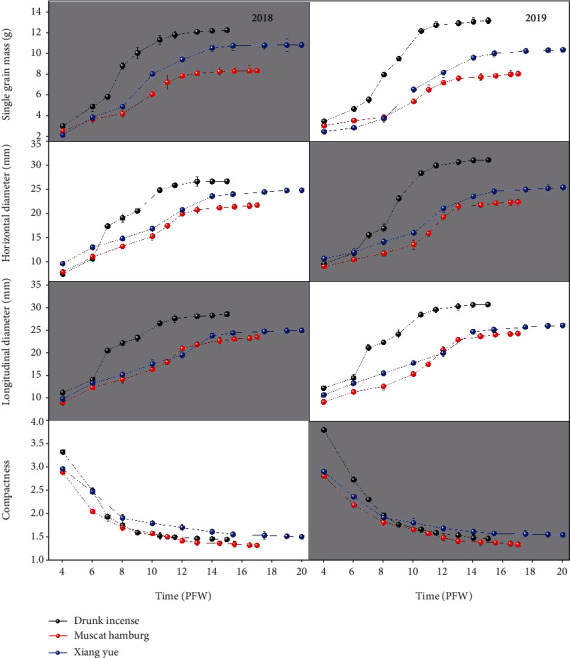
Changes in the physical characteristics of the three grape varieties at different postflowering weeks (PFW).

The growth of grape fruit usually begins about four weeks after flowering. The research started from the 4th week after flowering to monitor the physical and chemical indicators of the grapes. The dynamic changes in the physical characteristics of the three grape varieties (Drunk Incense, Muscat Hamburg, and Xiang Yue) in 2018 and 2019 are shown in Figure 2. In 2018, the single-grain weight of the three grape varieties (Drunk Incense, Muscat Hamburg, and Xiang Yue) all showed a slow-fast-slow growth trend; the growth curve of the single-grain weight of the three varieties in 2019 was similar to that in 2018, but the final single-grain weights of Drunk Incense, Muscat Hamburg, and Xiang Yue in 2019 reached 14.0 g, 8.54 g, and 11.02 g, respectively, which were 14.38%, 2.41%, and 1.85% higher, respectively, than those in 2018. The increase in single-grain weight may be due to the improved greenhouse production.

The growth of the longitudinal and horizontal diameters of grapes was similar, and the growth of both diameters was highly synchronized. In 2018 and 2019, the longitudinal diameters of Drunk Incense, Muscat Hamburg, and Xiang Yue reached 28.75 and 31.03 mm, 23.69 and 24.59 mm, and 25.17 and 26.39 mm, respectively. The compactness and fruit diameter of the three grape varieties showed the opposite trend, showing a trend of rapid decline first followed by essentially stable growth. The final compactness of Xiang Yue was the largest and was stable at approximately 1.55 in 2018 and 2019. The compactness of Muscat Hamburg and Drunk Incense grapes was approximately 1.45, with little interannual differences. In general, the relationship between the single-grain weight and horizontal diameter of the three grape varieties was the same, showing a slow-fast-slow increasing trend. The final single-grain weight and horizontal and longitudinal diameters were in the order of Drunk Incense > Xiang Yue > Muscat Hamburg. The compactness of the three grape varieties showed a gradual decreasing trend, and the final result was as follows: Xiang Yue > Drunk Incense > Muscat Hamburg. There were obvious differences in the type, weight, and compactness of the three kinds of grapes, and these differences were mainly related to the differences in varieties.

**Figure 3:**
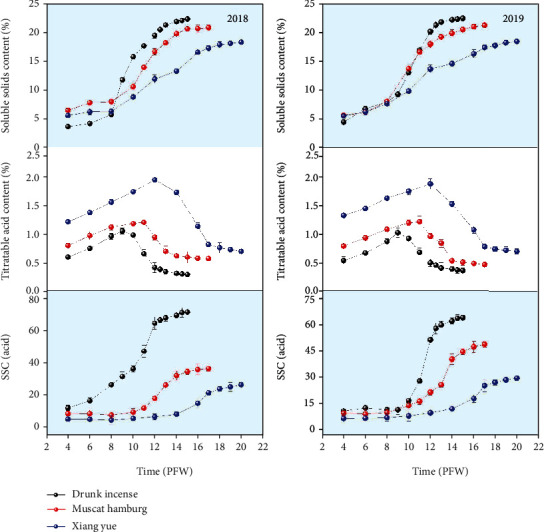
Dynamic changes of quality characteristics such as soluble solid content (SSC), titratable acid content, and SSC/acid at different postflowering weeks (PFW) of the three grape varieties.

The variation in the chemical properties of the three varieties of grapes from four weeks after flowering to maturity is shown in Figure 3. It can be seen from this figure that during the fruit ripening process, the soluble solid content and SSC/acid were essentially synchronized, showing a “slow-fast-slow” change trend, but there were obvious differences in the values of these two chemical properties of the three varieties. In the first stage, each chemical property increased slowly, and the three varieties of grapes differed little at this stage; this stage lasted approximately 4 weeks for all three varieties, and the second stage was the rapid growth stage. The duration of this stage was the shortest for Drunk Incense, lasting only 4-5 weeks; this stage was longer for Muscat Hamburg and Xiang Yue than for Drunk Incense, which lasted approximately 6 weeks and 7 weeks, respectively, and the duration of the third stage was 3-4 weeks. In 2018, the final soluble solid content of Drunk Incense, Muscat Hamburg, and Xiang Yue reached 22.25, 20.75, and 18.23%, and the SSC/acid reached 71.16, 36.08, and 26.04.

In 2018, the titratable acid content of the three varieties showed a trend of increasing first and then decreasing. The titratable acid contents of Drunk Incense, Muscat Hamburg, and Xiang Yue reached their maximum values at 9 weeks, 11 weeks, and 12 weeks after flowering, respectively, which were 1.05%, 1.2%, and 1.93%, respectively; afterward, they showed a downward trend, and the final values were 0.3, 0.57, and 0.7, respectively. The chemical properties of grapes in 2019 were essentially the same as those in 2018, and the soluble solids of the three varieties ultimately were 22.45, 21.25, and 18.4%. The final SSC/acid of Drunk Incense, Muscat Hamburg, and Xiang Yue were 60, 45.21, and 26.67, respectively; the titratable acid contents reached 0.368, 0.47, and 0.69%, respectively; and the final chemical properties had little interannual differences. The two-year data showed that the final chemical properties of the three varieties were consistent. The soluble solids and SSC/acid showed a gradual increasing trend of “slow-fast-slow,” and the titratable acid showed a trend of increasing first and then decreasing.

In general, the two-year data showed that beginning at 4 weeks after flowering to maturity, the horizontal and longitudinal diameter, single-grain weight, soluble solid content, and SSC/acid of the grapes all showed an increasing trend, while the compactness was the opposite, showing a decreasing trend. The titratable acid content shows a trend of first increasing and then decreasing. The three varieties of grapes exhibited similar changes in physical and chemical properties, but their change rates and final values were quite different. In addition, the horizontal and vertical diameters and the single-grain weight of the various grape varieties in 2019 were significantly higher than those in 2018, which may be related to the interannual differences in the greenhouse grape planting period and in the greenhouse environment in 2019.

### 3.2. Determination of the Grape Maturity Coefficient Based on the Physical and Chemical Properties of Grapes

**Figure 4:**
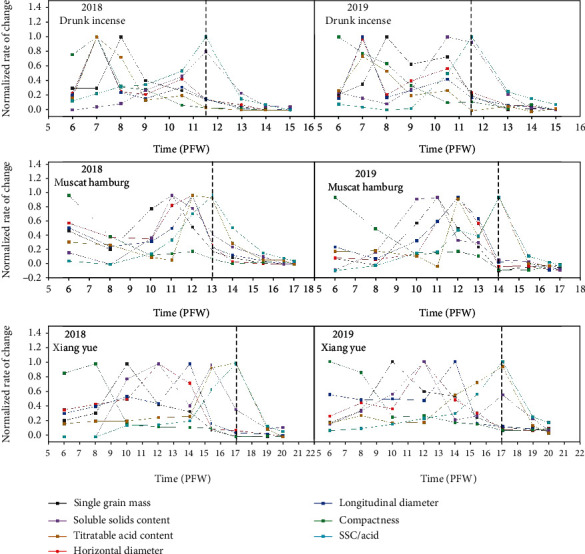
Normalized change rate of physical and quality properties of grape varieties with different postflowering weeks (PFW).

The physical and chemical indicators of the three varieties of grapes during different weeks after flowering were different in terms of their rate of change (Figures 2 and 3). As such, the indicators in different weeks after flowering were normalized, and each normalized value was then obtained. The rate of change of the normalized indicators is shown in Figure 4.

**Figure 5:**
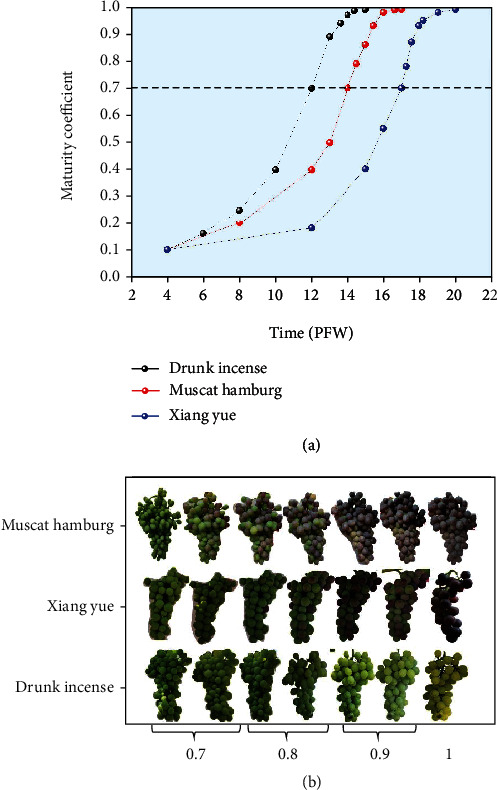
(a) Dynamic changes in maturity coefficients of the three varieties at different postflowering weeks (PFW). (b) Relationships between maturity images and maturity coefficients of grape varieties.

The two-year data showed that the change rate of the normalized indicators of the physical and chemical properties of Drunk Incense from 6 weeks after flowering to 11 weeks after flowering was relatively steep (Figure 4). After 11 weeks after flowering, the normalized rate of change of the soluble solid content and SSC/acid of Drunk Incense decreased significantly, and the normalized rate of change of the other indicators gradually tended toward zero. For Muscat Hamburg and Xiang Yue, the normalized rate of change of each index showed irregularity at 6-14 and 6-17 weeks after flowering, and the rate became more intense. However, after 14 and 17 weeks, the normalized titratable acid content and SSC/acid of these two grape varieties also decreased rapidly and gradually tended toward zero. According to the above-mentioned physical and chemical indicator changes at different stages of grape ripening, the ripening process of the grapes could be divided into two stages: an immature stage and a mature stage. The first stage is the immature stage. For the three varieties, the first stage starts at 4 weeks after flowering and progresses until 12 weeks (Drunk Incense), 14 weeks (Muscat Hamburg), and 17 weeks after flowering (Xiang Yue). During this stage, the physical and chemical properties of the three varieties of grapes all changed drastically; the second stage is the fruit color change and ripening period, and the durations of this stage for the three varieties were 12-15 weeks after flowering (Drunk Incense), 14-17 weeks (Muscat Hamburg), and 17-20 weeks (Xiang Yue). During this stage, the normalized rate of change of various chemical indicators of grapes decreased significantly, and the rate of change of other indicators gradually tended toward 0. The Mc was used to quantitatively describe the ripening process of grapes. When the Mc was between 0.1 and 0.7, the grapes were immature (the growth stages of Muscat Hamburg, Drunk Incense, and Xiang Yue were 4-12 weeks, 4-14 weeks, and 4-17 weeks after flowering, respectively). When the Mc was between 0.7 and 1, the grapes gradually matured; 0.7 represents partially mature grapes, and 1 represents fully mature grapes (the normalized rate of change of all indicators was less than 0.2). The dynamic changes in grape maturity coefficients during different weeks postflowering are shown in Figure 5(a). It can be clearly seen from this figure that the Mc values of Drunk Incense, Muscat Hamburg, and Xiang Yue grapes showed a “slow-fast-slow” trend during fruit development and maturation. However, there were obvious differences in the maturity cycle; they reached partial maturity at approximately 12, 14, and 17 weeks after flowering (Mc = 0.7), which corresponded to the early, middle, and late stages of the maturity cycle, respectively. The corresponding relationship between the Mc values and the fruit images of the three varieties at the ripening stage is shown in Figure 5(b).

**Figure 6:**
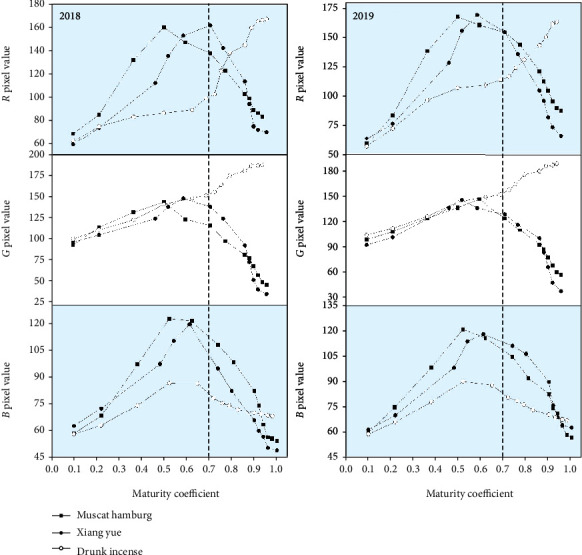
Changes in *R*, *G*, and *B* pixel values in the growth and development of the three grape varieties.

**Figure 7:**
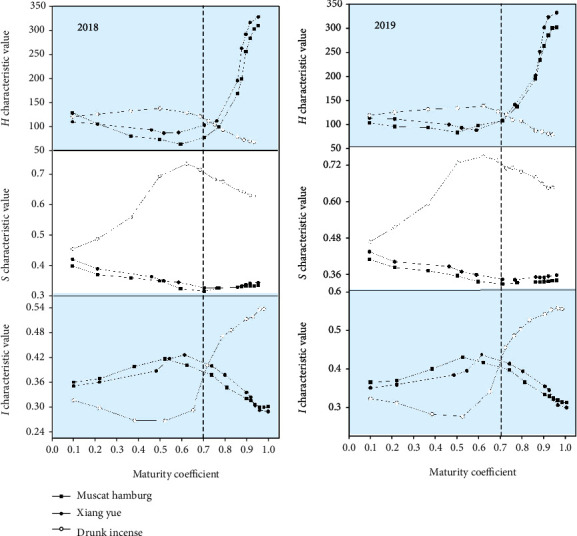
Changes in *H*, *S*, and *I* values during the growth and development of the three grape varieties.

Machine vision technology was used to collect the pixel data (*R*, *G*, and *B*) of the grape images, and the *H*, *I*, and *S* color values were calculated according to Formulas (1)-(5). The changes in the RGB color values and HSI values of the three grape varieties during the whole growth period are shown in Figures 6 and 7.  Figure 6 shows that during the development of the grapes, the *R* pixel values of the epidermis of Muscat Hamburg and Xiang Yue grapes differed a little, and both show a single peak change. The Muscat Hamburg peak supersedes the Xiang Yue peak by 160.13, and the decreasing trend occurs almost simultaneously. The change in *R* value of Drunk Incense is different from that of the previous two varieties, showing a steady increasing trend. Moreover, the change trends of the *G* values and *R* values of the grape epidermis of the Muscat Hamburg and Xiang Yue varieties are essentially the same—both show a single peak change trend. However, the *G* value of the Drunk Incense variety of the epidermis increases steadily throughout the growth process, and the *B* values of the three varieties of grapes exhibited essentially the same change in the epidermis, showing a trend of first increasing and then decreasing. The *B* values of the Muscat Hamburg and Xiang Yue grape epidermis are essentially the same, and the *B* values are obviously relatively low.

It can be seen from Figure 7 that the *H*, *S*, and *I* values of the grape skins of the Muscat Hamburg and Xiang Yue varieties are essentially synchronized with the change trend of the maturity coefficient; these values first decrease and then increase, gradually decrease, and first increase and then decrease, respectively. The *H*, *S* and *I* values of the skin of Drunk Incense grape are opposite the color values of the other two kinds of grapes; these values first increase and then decrease, first decrease and then increase, and gradually increase, respectively. The RGB pixel values and HSI color values of the three grape varieties vary with maturity coefficient. However, the overall performance of Muscat Hamburg and Xiang Yue is essentially similar. This may cause some interference in the prediction of grape maturity. Except for the *B* value Drunk Incense grapes, the change trend of the other two varieties is not much different, but the change trend of other pixel values and color values is obvious.

### 3.3. Prediction of the Maturity of Greenhouse-Grown Grapes

According to the above research, the number of weeks postflowering can be used to roughly estimate the ripening status of grapes. To further accurately determine the ripening status of grapes, the color value of the grape skin was used to predict the maturity of the grapes during the ripening period. The color feature values (*R*, *G*, *B*, *H*, *S*, and *I*) were used to predict the maturity of the three grape varieties, and the predictive effects are shown in Table 1. It can be seen from the table that the coefficient of determination (*R*^2^) of each color value for the maturity prediction of the three varieties of grapes was between 0.36 and 0.65 (*P* < 0.05). For Drunk Incense grape, the *R* value prediction accuracy was the highest: the *R*^2^ of the prediction model reached 0.65, the MAE and RMSE were only 0.09 and 0.12, respectively, and the *d* value was 0.76. The prediction accuracy of the *G* and *I* models was also good, of which the *R*^2^, MAE, and RMSE of the *G* model reached 0.63, 0.1, and 0.13, respectively, which were better than those of the *I* model; however, the consistency index (*d*) of the *G* model was 0.7, which was slightly lower than the 0.73 of the *I* model. The GPI evaluation index was used to comprehensively evaluate the indicators of each prediction model [13]. The three models with greatest evaluation scores were the *R*, *G*, and *I* models, and their comprehensive evaluation scores were 0.87, 0.39, and 0.14, respectively. For the Muscat Hamburg *G* index model, the *R*^2^ was the highest, reaching 0.61, but the *d* of the *H* model was 0.73. The GPI comprehensive index revealed that the three models with the best simulation results were the *H*, *G*, and *I* models. For Xiang Yue, after the comprehensive GPI-based evaluation, the three models with the best simulation results were the *H*, *I*, and *G* models. In general, although the best single-factor prediction models were different for different varieties of grapes, the *R*, *G*, *H*, and *I* models were all single-factor prediction models with prediction effects better than those of two-factor models.

**Figure 8:**
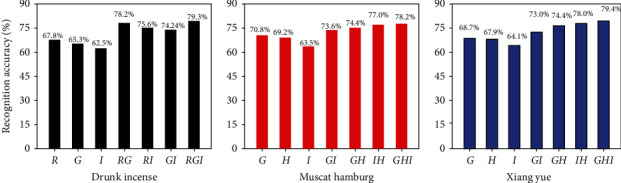
Recognition accuracy of different color feature value combinations based on BPNN.

To further improve the accuracy of grape maturity evaluation, this study used three single-factor models with higher prediction accuracy for each variety to construct a multifactor prediction model. For Drunk Incense, the seven models were *R*, *G*, *I*, RG, RI, GI and RGI; for Muscat Hamburg and Xiang Yue, seven other models (*G*, *I*, *H*, GI, GH, IH, and GIH) were used to make predictions. For the two-factor and three-factor models, a back-propagation neural network was used to predict the whole growth of the three grape varieties. The number of nodes in the input layer of the BPNN was consistent with the corresponding number of features. The transfer function of the layer and the hidden layer was a tangent *S*-type function, and the transfer function of the output layer was a linear function. The recognition result is shown in Figure 8. It can be seen from the figure that the use of the two-factor combination model for different grape varieties improved the prediction accuracy of grape maturity to varying degrees. Compared with the accuracy of its the single-factor model (model *I*), that of Drunk Incense's two-factor model increased by approximately 15.7%. Moreover, compared with that of the three-factor *RGI* model, the accuracy the optimal two-factor *GI* model was further improved by 1.1%, although the improvement was not large; Muscat Hamburg and Drunk Incense also showed similar results. The two-factor model had significantly improved accuracy compared with that of the single-factor model, but the optimal two-factor model's accuracy was not much different from that of the three-factor model; moreover, the addition factor significantly increased the complexity of the model. Therefore, the GI, HI, and HI models were selected as the best models for judging the maturity coefficient of Drunk Incense, Muscat Hamburg, and Xiang Yue.

Prediction of the grape maturity coefficient can lead to improved management of grapes. When the grapes were in the ripening period (during which the maturity coefficient was between 0.7 and 1), the grapes gradually matured, and the difference in physical indicators was small. Accurate determination was crucial for accurately determining the prime time for grape picking. Based on a BPNN, a two-factor model with relatively high prediction accuracy was used to predict the maturity coefficient of grapes during the color-changing ripening period. The corresponding relationships between the maturity image of each grape variety and the maturity coefficient are shown in Figure 3(b).

The three varieties of grapes were judged at the ripening period. As shown in Table 2, in the ripening period, when the optimal model was used, the accuracy of judging the ripening period of the three varieties was between 76.0% and 88.0%. Among them, the judgment accuracy of Xiang Yue was the best, with an accuracy rate of 88.0%. The overestimation rate and the underestimation rate were only 6.7% and 5.3%, respectively, followed by Muscat Hamburg and Drunk Incense, which had the lowest overestimation rate, and the accuracy rate of discrimination was 76.0%.

**Figure 9:**
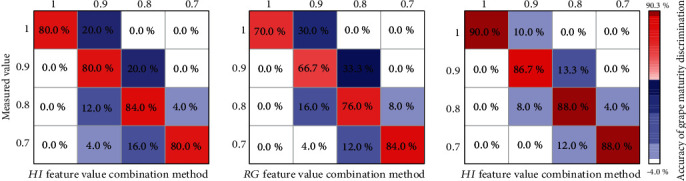
Measured versus predicted grape maturity of the three varieties during the growing season.

Using the confusion matrix, the maturity of the three grape varieties (Drunk Incense, Muscat Hamburg, and Xiang Yue) was judged separately (when the Mc was 0.7, 0.8, 0.9, and 1, the sampling number was divided into 25, 25, 15, and 10, respectively; the total sampling number of each variety was 75 bunches of grapes).  Figure 9 shows that the RG and HI combination method was used to judge the maturity of the three varieties (Muscat Hamburg, Drunk Incense, and Xiang Yue). When the maturity of the three varieties was 1, the accuracy of Xiang Yue's predictive ability was the highest. The accuracy rate of Muscat Hamburg was 90.0%, followed by that of Muscat Hamburg, which was 80.0%. Drunk Incense had the lowest accuracy rate of 70.0%. When the maturity coefficient was 0.9, the accuracy rates of the three grape varieties (Muscat Hamburg, Drunk Incense, and Xiang Yue) were 80.0%, 66.7%, and 86.7%, respectively. Xiang Yue's prediction accuracy was the highest, Muscat Hamburg's was the second highest, and Drunk Incense's was the lowest, at only 66.7%. When Drunk Incense grapes were mature (Mc of 0.9), the prediction error with this method was relatively large, and the judgment was underestimated 33.3%. The prediction accuracy of the three varieties (Muscat Hamburg, Drunk Incense, and Xiang Yue) was also relatively high, reaching 80.0%, 84.0%, and 88.0%, respectively, at an Mc value of 0.7 and 84.0% and 76.0% and 88.0%, respectively, at an Mc values 0.8. The prediction accuracy for the three varieties was in the following order: Xiang Yue > Muscat Hamburg > Drunk Incense. In addition, at different maturity levels, the model had different prediction accuracies for the three varieties. Muscat Hamburg had the highest prediction accuracy when it was mature (84.0%), and Drunk Incense had the highest prediction accuracy when its Mc value was 0.7 (84.0%). The accuracy of judging Xiang Yue grapes was the highest (90.0%) when its Mc value was 0.9. In addition, Figure 9 also shows that when the grapes presented an Mc value of 0.9 or were fully mature, the different methods underestimated the maturity of the grapes by a certain percentage. At an Mc of 0.8, overestimation and underestimation were likely to occur, especially for Muscat Hamburg and Drunk Incense. At an Mc of 0.8, the proportions of overestimation and underestimation reached 12.0% and 4.0% and 16.0% and 8.0%, respectively. To reduce the damage to the grapes, the number of grape spikes sampled at each maturity level of the same variety was different, so this study did not compare the prediction accuracy of the same variety under different maturity levels.

## 4. Discussion

The prediction accuracy (*R*^2^) values of Drunk Incense, Muscat Hamburg, and Xiang Yue using the *H* value were 0.56, 0.58, and 0.59, respectively. These prediction accuracies were not good, and they were lower than those of recent studies that used the *H* value to predict tomato maturity (*R*^2^ up to 99.3%) [31]. This may be due to the difference in the color values of *H* in different pericarps. The color of tomato skin changed from green to orange and then to red, and the value of *H* tended to steadily decrease, changing obviously during the whole growth period. Grape skins, on the other hand, changed from green to purple, pink, or a yellowish color. The *H* value of the epidermis of Muscat Hamburg and Xiang Yue grapes tended to steadily increase during the color-changing maturity period (Figure 7), and the change was obvious throughout the whole growth period, so the prediction accuracy was relatively high. However, the RH value of the Drunk Incense grape variety during the whole growth period increased first and then decreased; the variation trend was not obvious, so the prediction accuracy was the lowest. The color changes of the grape skins were similar to results in a previous study [30]. In addition, the results when the *G* value was used to determine the maturity of the three grape varieties were all less than 0.63, and these results were similar to those of a previous study [33]. In the RGB color space, the *R*, *G*, and *B* values are highly correlated, and RGB pixel values are easily affected by light conditions in an open environment [34]; therefore, it is difficult to predict grape maturity when RGB color feature values are used. However, unlike us, Pereira et al. [28] used the normalized average value of the RGB color space to predict the maturity of papaya; the effect was better, and the accuracy rate was 78.1%. The color of the papaya skin changes mainly from green to yellow, and the color change is relatively simple, which may be the main reason explaining their results.

In this study, Drunk Incense's two-factor model (RG model) improved the prediction accuracy by approximately 15.7% compared to that of the single-factor model (*I* model). When using the HI model to predict the maturity coefficient of Muscat Hamburg and Xiang Yue, this model was better than single-factor model (*I* model), and the prediction accuracy increased by 13.5% and 13.9%, respectively. However, when the three-factor RGI and GHI models were used to predict the maturity coefficients of Drunk Incense, Muscat Hamburg, and Xiang Yue, the maturity coefficients increased by 1.1%, 1.2%, and 1.4%, respectively, compared with the highest accuracies achieved with the two-factor RG and HI models. The two-factor models could predict the maturity of different grapes, and the prediction accuracy was significantly higher than that of the single-factor models. However, the accuracy of the three-factor models was not significantly improved. These findings were similar to those of the previous studies that used machine learning models and digital images to predict fresh spinach stages [35]. The results of the present study showed that the more combination factors there were, the lower the accuracy of the model and the overall prediction effect. The reason can be explained by probability theory; in the same random sampling method, the fewer the number of factors, the easier it is for accuracy to increase.

In addition, previous studies have also pointed out that using an image segmentation algorithm based on a BPNN to classify mangoes was more accurate than existing methods [36]. Based on the BPNN model, using the combined information of two color channels to predict the soluble solids of apples was better than using a single-channel model, which was consistent with the research results in this paper [37]. From the analysis of the confusion matrix of grape maturity judgment, it can be concluded that the accuracy of the two-factor combination method for the judgment of grape maturity was the best; specifically, the ability of this model was best for Xiang Yue, followed by Muscat Hamburg and then Drunk Incense (Figure 9). The reason for these results may be due to the different trends and degrees of change of the three varieties during the color conversion and maturity periods. For Drunk Incense, the skin color of grapes on the same bunch is relatively similar throughout the growth process, and the judgment error mainly occurs between Mc values of 0.8 and 0.9 [38]. For example, Ponce et al. [39] also found that due to the different cherry fruit varieties, differences in the accumulation of anthocyanins during the fruit development process led to potential differences in fruit evolution, similar to the results of our study.

## 5. Conclusion

The two-year data showed that the fruit development process of Drunk Incense, Muscat Hamburg, and Xiang Yue grapes showed a “fast-slow-fast” change trend from 4 weeks after flowering to maturity. According to the comparative analysis of the normalized rate of change of the physical and chemical indicators of the three varieties, the maturity coefficient (Mc) of the three varieties was determined, the relationship between the maturity coefficient and the postflowering cycle was determined, and Drunk Incense and Muscat Hamburg were clarified. The demarcation points of the immature and color-changing ripening periods of Drunk Incense, Muscat Hamburg, and Xiang Yue grapes were 14, 15, and 17 weeks after flowering (Mc = 0.7). Through the determination of grape skin color values (*R*, *G*, *B*, *H*, *S*, and *I*), it was found that there was little difference between the *R*, *G*, and *B* and *H*, *I*, and *S* changes of Muscat Hamburg and Xiang Yue, but the difference between these for Drunk Incense was obvious. Using the above single factors to construct a grape maturity prediction model, the best prediction indicators of the different varieties were inconsistent, but the *R*, *G*, *H*, and *I* models were all single-factor prediction models with better prediction effects than those of the other models. Using two factors can significantly improve the prediction accuracy of the models, but the accuracy of the three-factor models did not significantly improve. The best two-factor prediction models for Muscat Hamburg, Drunk Incense, and Xiang Yue were the HI, RG, and HI models, respectively, with prediction accuracies of 77.0%, 78.2%, and 78.0%, respectively. Using a better two-factor model to compare the prediction accuracy of Muscat Hamburg, Drunk Incense, and Xiang Yue's color conversion maturity period, the prediction accuracy of Xiang Yue was the highest (88.0%), followed by Muscat Hamburg; the accuracy of Drunk Incense was the lowest (only 76.0%). In addition, there were also differences in the prediction accuracy of different grape varieties at each maturity level. Muscat Hamburg, Drunk Incense, and Xiang Yue had the highest prediction accuracies at Mc values of 0.7, 0.8, and 0.9, respectively, and the accuracy of judgment reached 84.0%, 84.0%, and 90.0%, respectively.

## Figures and Tables

**Table 1:** Predictive effect of the three varieties of grape maturity.

Variety	Statistical indicators	Color feature value					
		*R*	*G*	*B*	*H*	*S*	*I*
Drunk incense	*R* ^2^	0.65	0.63	0.41	0.56	0.37	0.61
	MAE	0.09	0.1	0.11	0.12	0.18	0.12
	RMSE	0.12	0.13	0.24	0.14	0.26	0.13
	*d*	0.76	0.7	0.67	0.72	0.62	0.73
	GPI	0.87	0.39	-2.448	-0.216	-3.03	0.142
	GPI rank	1	2	5	4	6	3

Muscat Hamburg	*R* ^2^	0.36	0.61	0.48	0.58	0.41	0.58
	MAE	0.24	0.1	0.14	0.16	0.17	0.11
	RMSE	0.29	0.12	0.13	0.13	0.25	0.16
	*d*	0.53	0.75	0.68	0.73	0.59	0.71
	GPI	-2.915	0.855	-0.653	1.085	-1.707	0.653
	GPI rank	6	2	4	1	5	3

Xiang Yue	*R* ^2^	0.38	0.6	0.42	0.59	0.46	0.57
	MAE	0.23	0.08	0.1	0.09	0.16	0.13
	RMSE	0.27	0.11	0.23	0.12	0.11	0.12
	*d*	0.51	0.61	0.54	0.6	0.54	0.59
	GPI	-2.683	0.785	-1.484	1.317	-0.776	1.042
	GPI rank	6	3	5	1	4	2

**Table 2:** Comparison of the prediction accuracy of the maturity coefficients of the three varieties of grapes during the color transition period.

Variety	Prediction model	Total number of samples	Correct number of samples	Number of overestimated number of samples	Number of underestimated samples	Accuracy rate	Overestimation rate	Underestimation rate
Muscat Hamburg	RG	75	61	7	6	81.3%	9.3%	8%
Drunk incense	HI	75	57	8	10	76%	10.7%	13.3%
Xiang Yue	HI	75	66	5	4	88%	6.7%	5.3%

## Data Availability

The data could be given upon reasonable request from the corresponding author.

## References

[1] Anderson N. T., Walsh K. B., Wulfsohn D. (2021). Technologies for forecasting tree fruit load and harvest timing—from ground, sky and time. *Agronomy*.

[2] Zhao C. (2014). Advances in agricultural remote sensing research and application. *Journal of Agricultural Machinery*.

[3] Van Beers R., Aernouts B., Gutiérrez L. L. (2015). Optimal illumination-detection distance and detector size for predicting Braeburn apple maturity from Vis/NIR laser reflectance measurements. *Food and Bioprocess Technology*.

[4] Liu T., Yuan Q., Wang Y. (2021). Hierarchical optimization control based on crop growth model for greenhouse light environment. *Computers and Electronics in Agriculture*.

[5] Lin F., Weng Y., Chen H., Zhuang P. (2021). Intelligent greenhouse system based on remote sensing images and machine learning promotes the efficiency of agricultural economic growth. *Environmental Technology & Innovation*.

[6] Du J., Lu X., Fan J., Qin Y., Yang X., Guo X. (2020). Image-based high-throughput detection and phenotype evaluation method for multiple lettuce varieties. *Frontiers in Plant Science*.

[7] Sun G., Wang X., Sun Y., Ding Y., Lu W. (2019). Measurement method based on multispectral three-dimensional imaging for the chlorophyll contents of greenhouse tomato plants. *Sensors*.

[8] Zhuo W., Huang J., Gao X. (2020). Prediction of winter wheat maturity dates through assimilating remotely sensed leaf area index into crop growth model. *Remote Sensing*.

[9] Minas I. S., Blanco-Cipollone F., Sterle D. (2021). Accurate non-destructive prediction of peach fruit internal quality and physiological maturity with a single scan using near infrared spectroscopy. *Food Chemistry*.

[10] Prayogi I. Y., Damayanti R., Djoyowasito G. (2020). Design to prediction tools for banana maturity based on image processing. *Conference Series: Earth and Environmental Science*.

[11] Jiang Y. P., Chen S. F., Bian B., Li Y., Sun Y., Wang X. (2021). Discrimination of tomato maturity using hyperspectral imaging combined with graph-based semi-supervised method considering class probability information. *Food Analytical Methods*.

[12] Xu S., Li J., Baldwin E. A. (2018). Electronic tongue discrimination of four tomato cultivars harvested at six maturities and exposed to blanching and refrigeration treatments. *Postharvest Biology and Technology*.

[13] Gao Q., Wang P., Niu T. (2022). Soluble solid content and firmness index assessment and maturity discrimination of Malus micromalus Makino based on near-infrared hyperspectral imaging. *Food Chemistry*.

[14] Jie D. F., Wu S., Wang P., Li Y., Ye D., Wei X. (2021). Research on Citrus grandis granulation determination based on hyperspectral imaging through deep learning. *Food Analytical Methods*.

[15] Shah Z., Raja M. A. Z., Chu Y. M. (2021). Computational intelligence of Levenberg-Marquardt backpropagation neural networks to study the dynamics of expanding/contracting cylinder for cross magneto-nanofluid flow model. *Physica Scripta*.

[16] Sun S., Liang N., Zuo Z. (2021). Estimation of botanical composition in mixed clover–grass fields using machine learning-based image analysis. *Frontiers in Plant Science*.

[17] Huang J., Luo X., Peng X. Y. (2020). A novel classification method for a driver's cognitive stress level by transferring interbeat intervals of the ECG signal to pictures. *Sensors*.

[18] Olawoyin R. (2016). Application of backpropagation artificial neural network prediction model for the PAH bioremediation of polluted soil. *Chemosphere*.

[19] Wang X. H., Yuan J., Wang B. Z. (2021). Prediction and analysis of PM2.5 in Fuling District of Chongqing by artificial neural network. *Neural Computing & Applications*.

[20] El-Bendary N., El Hariri E., Hassanien A. E., Badr A. (2015). Using machine learning techniques for evaluating tomato ripeness. *Expert Systems with Applications*.

[21] Xiong J. T., Zou X. J., Liu N., Peng H. X., Li J. H., Lin G. C. (2014). Quality detection technology of litchi fruit during picking based on machine vision. *Journal of Agricultural Machinery*.

[22] Liu G. M., Zou M., Liu M. H., Li J. (2008). Preliminary study on computer vision inspection technology of navel orange external quality. *China Agricultural Science and Technology Guide*.

[23] Harel B., van Essen R., Parmet Y., Edan Y. (2020). Viewpoint analysis for maturity classification of sweet peppers. *Sensors*.

[24] Vrochidou E., Bazinas C., Manios M., Papakostas G. A., Pachidis T. P., Kaburlasos V. G. (2021). Machine vision for ripeness estimation in viticulture automation. *Horticulturae*.

[25] Liu X., Li Z., Zhang L., Liu Y., Li Y., Li T. (2021). Effect of single tube sections on the structural safety of Chinese solar greenhouse skeletons. *Scientific Reports*.

[26] Su Z., Zhang C., Yan T. (2021). Application of hyperspectral imaging for maturity and soluble solids content determination of strawberry with deep learning approaches. *Frontiers in Plant Science*.

[27] Meng R., Wang Y. J., Zhang B. H., Wu Y. N., Yang Y. Z., Zhao Z. Y. (2015). Changes of anthocyanin synthesis in peel of ‘Granny Smith’fruit after bagging. *Food Science*.

[28] Pereira L. F. S., Barbon S., Valous N. A., Barbin D. F. (2018). Predicting the ripening of papaya fruit with digital imaging and random forests. *Computers and Electronics in Agriculture*.

[29] Veluchamy M., Subramani B. (2020). Fuzzy dissimilarity color histogram equalization for contrast enhancement and color correction. *Applied Soft Computing*.

[30] Chen B., Shi S., Sun J. (2021). Using HSI color space to improve the multispectral lidar classification error caused by measurement geometry. *IEEE Transactions on Geoscience and Remote Sensing*.

[31] Wan P., Toudeshki A., Tan H., Ehsani R. (2018). A methodology for fresh tomato maturity detection using computer vision. *Computers and Electronics in Agriculture*.

[32] Li B., Ge D., Wei X. G., Zheng S. Y., Sun J., Yang X. Y. (2020). High-precision extraction of grape green fraction in solar greenhouse based on subsection extraction method. *Journal of Shenyang Agricultural University*.

[33] Liu T. H., Ehsani R., Toudeshki A., Zou X. J., Wang H. J. (2019). Identifying immature and mature pomelo fruits in trees by elliptical model fitting in the Cr–Cb color space. *Precision Agriculture*.

[34] Tan K., Lee W. S., Gan H., Wang S. (2018). Recognising blueberry fruit of different maturity using histogram oriented gradients and colour features in outdoor scenes. *Biosystems Engineering*.

[35] Koyama K., Tanaka M., Cho B. H., Yoshikawa Y., Koseki S. (2021). Predicting sensory evaluation of spinach freshness using machine learning model and digital images. *PLoS One*.

[36] Kumari N., Bhatt A. K., Dwivedi R. K., Belwal R. (2021). Hybridized approach of image segmentation in classification of fruit mango using BPNN and discriminant analyzer. *Multimedia Tools and Applications*.

[37] Huang Y., Wang J., Li N., Yang J., Ren Z. (2021). Predicting soluble solids content in “Fuji” apples of different ripening stages based on multiple information fusion. *Pattern Recognition Letters*.

[38] Yang S., Feng Q., Wang S. Z., Zhang R. (2017). Detection and tracking of grape leaves based on improved deformable component model and discriminant model. *Journal of Agricultural Engineering*.

[39] Ponce C., Kuhn N., Arellano M. (2021). Differential phenolic compounds and hormone accumulation patterns between early-and mid-maturing sweet cherry (Prunus avium L.) cultivars during fruit development and ripening. *Journal of Agricultural and Food Chemistry*.

